# Modeling diet-gut microbiome interactions and prebiotic responses in Thai adults

**DOI:** 10.1038/s41522-026-00921-z

**Published:** 2026-01-28

**Authors:** Nachon Raethong, Preecha Patumcharoenpol, Wanwipa Vongsangnak

**Affiliations:** 1 Institute of Nutrition, Mahidol University, Nakhon Pathom, Thailand; 2 Division of Medical Bioinformatics, Research Department, Faculty of Medicine Siriraj Hospital, Mahidol University, Bangkok, Thailand; 3 Department of Zoology, Faculty of Science, Kasetsart University, Bangkok, Thailand

**Keywords:** Microbial communities, Microbiome

## Abstract

The impact of diet on gut microbial metabolism is essential for advancing microbiome-based health interventions. This study introduces a novel systems biology pipeline that integrates genome-scale metabolic models (GSMMs) with Thai dietary intake data to simulate gut microbiome metabolism and assess prebiotic responses. Utilizing metagenomic data from healthy Thai adults and an average Thai diet derived from national surveys, community-scale metabolic models (CSMMs) were developed and simulated under both typical dietary and prebiotic-supplemented condition. Flux variability analysis was employed to assess metabolic capacities, short-chain fatty acids (SCFAs) production in relation to microbial taxonomy. The results promisingly revealed inter-individual variability in SCFA profiles, with *Bacteroides* and *Phocaeicola* notably linked to isobutyrate production and *Bifidobacterium* emerged as a key responder to prebiotic supplementation. This integrative framework offers biological insights into diet-gut microbiome interactions and provides a foundation for the development of precision nutrition strategies tailored to the Thai population.

## Introduction

Diet is a key component of the relationship between gut health and microbiome^1^. The gut microbiome responses to dietary interventions, such as probiotics, prebiotics, and fiber-rich foods, demonstrated impacts on human physiology, particularly through the complex exchange of metabolites, e.g., short-chain fatty acids (SCFAs)^2^. Understanding trophic interactions in diet, gut health, and microbiome is essential for developing microbiome-based dietary strategies as alternatives to conventional drug therapies^3^. However, assessing gut microbiome responses to dietary interventions remains a significant challenge due to nonlinear microbial dynamics in response to diet and individual host variability.

Metagenomic and metatranscriptomic approaches have improved our ability to infer the compositions and metabolic functions of the gut microbiome^4–6^. However, these techniques are typically limited in their ability to capture metabolite exchanges^7,8^. Another approach is metabolomic analysis, which directly profiles metabolites produced by the gut microbiome under various conditions^9,10^. Nonetheless, an elucidating the mechanisms by which the microbiome influences the metabolome remains challenging, as statistical associations may result from indirect effects and confounding factors^11,12^. Alongside advancements in systems biology, genome-scale metabolic models (GSMMs) have enabled the assessment of the overall metabolism of hundreds of species within the human gut ecosystem^13^. Constraint-based modeling has been developed and applied using GSMMs to analyze metabolite exchange networks within the community^14,15^. This modeling approach offers valuable insights into host-gut microbiome interactions during early life^16^. To date, the modeling of gut microbiome metabolism has been primarily used to study human diseases, e.g., inflammatory bowel diseases^17,18^ and Alzheimer’s disease^19^. Existing studies often focus on Western populations and lack a systematic approach to integrating gut microbiome metabolism with alternative dietary influences. In Thai population, microbiome modeling research is still limited, despite the unique dietary patterns and cultural practices. Traditional Thai diets, rich in herbs, fermented foods, and complex carbohydrates, elicit distinct gut microbiome responses compared to Western dietary patterns^20^.

In this study, we present an integrated diet-gut microbiome modeling pipeline aimed at evaluating how the average Thai diet and prebiotic interventions influence gut microbial metabolism in Thai adults. Using data from the national food consumption survey (FCS)^21^ and Thai food composition database (FCD)^22^, the average Thai diet was translated into metabolite constraints for model simulations. Gut microbiome profiles of healthy Thai adults were mapped to GSMMs to construct community-scale metabolic models (CSMMs), which were contextualized under Thai dietary and prebiotic-supplemented conditions. Flux variability analysis (FVA) was then performed to estimate metabolite secretion potentials and target microbial taxon contributions. This approach successfully identified microbial responders to nutrients and prebiotics as well as provided biological insights into diet-gut microbiome interactions.

## Results

### An integrated diet-gut microbiome modeling pipeline

**Fig. 1:**
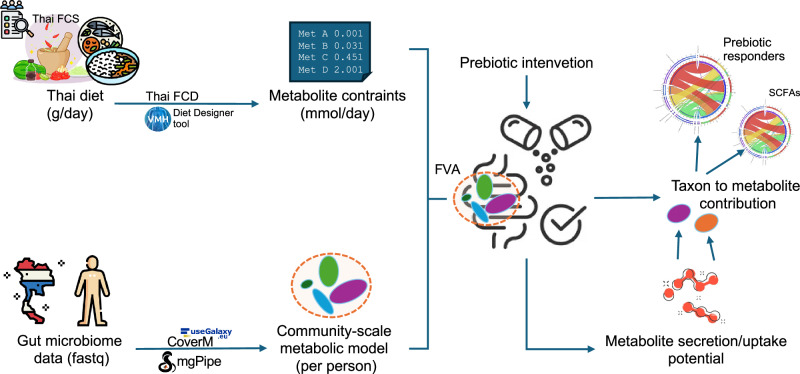
Systematic overview of an integrated diet-gut microbiome modeling pipeline.

The integrated diet-gut microbiome modeling pipeline was designed to assess how the average Thai diet and prebiotic interventions influence gut microbial metabolism (Fig. 1). Thai dietary intake data, collected in grams per day (g/day) from national FCS^21^, were processed using the Thai FCD^22^ and the Virtual Metabolic Human (VMH) diet designer tool^23^ to calculate metabolite constraints in mmol/day. These constraints represented a comprehensive list of nutrients-derived metabolites that define the average Thai diet and served as the reference background for modeling the Thai gut microbiome. Concurrently, gut microbiome data from healthy Thai adults were quality-filtered and mapped to the GSMM reference. The resulting strain-level relative abundances for each sample were then used as input to construct CSMMs. The constructed CSMMs were contextualized with either the average Thai diet or a prebiotic intervention. FVA was conducted under the given metabolite constraints to estimate the secretion and uptake potentials of microbial metabolites within the simulated gut ecosystem. The contribution of microbial taxon to target metabolites was analyzed to identify SCFAs producers and prebiotic responders.

### Definition of an average Thai diet

Dietary constraints for the Thai diet were estimated based on the average daily intake of the 26 most commonly consumed food items among Thai individuals, as listed in Table 1. Among these, 20 food items corresponded to existing food composition data in the VMH database. Due to some traditional foods, including dumpling, pork meatball, cricket, Thai dessert (Khao Tom Mud), fish sauce, and chicken essence were not listed in the VMH database, food composition data from the Thai FCD were then used.

**Table 1:** List of the most consumed food items among Thai individuals

Food item	Average daily intake (g/day)	VHM database
Rice	262.53 ± 46.01	Matched
Dumpling	68.12 ± 12.58	Unmatched
Sandwich	60.12 ± 7.80	Matched
Sweet potato	69.12 ± 10.23	Matched
Peanuts	54.78 ± 7.50	Matched
Cowpeas	33.33 ± 5.41	Matched
Mushrooms	29.96 ± 4.63	Matched
Pickled cabbage	24.67 ± 4.07	Matched
Watermelon	202.27 ± 29.06	Matched
Snacks, banana chips	25.00 ± 4.20	Matched
Egg	55.42 ± 4.94	Matched
Pork	31.02 ± 5.45	Matched
Pork meatball	62.75 ± 11.53	Unmatched
Fish, tilapia	64.43 ± 12.60	Matched
Fish, mackerel	30.37 ± 5.23	Matched
Insect, cricket	22.26 ± 4.45	Unmatched
Milk	241.01 ± 32.73	Matched
Ice cream	62.36 ± 4.65	Matched
Beer	454.17 ± 301.01	Matched
Snacks, potato chips	22.88 ± 5.45	Matched
Thai dessert (Khao Tom Mud)	88.66 ± 11.42	Unmatched
Sugar	6.37 ± 1.11	Matched
Oil, palm	9.81 ± 1.22	Matched
Fish sauce	5.37 ± 0.78	Unmatched
Chicken essence	47.57 ± 4.87	Unmatched

By integrating from resource data, an average Thai diet was constructed, totally 2295 kcal, with approximate macronutrient contributions of 54.65% carbohydrates, 23.66% fats, and 21.69% proteins. When verified against the Thai Dietary Reference Intake 2020 (Thai DRI), the energy content of this average Thai diet coordinated within the recommended daily energy intake range of 1500–2400 kcal for the general Thai population aged 9–65 years^24^. This range considered variations based on sex, age, and physical activity level with the lower end corresponding to the needs of moderately active adult females and the upper end corresponding to the needs of physically active adolescent males, thereby encompassing the most age and lifestyle groups in Thailand. According to the Thai DRI, carbohydrates should contribute 45–65% of total daily energy intake across all age groups. The calculated carbohydrate content of 54.65% in the average Thai diet falls within this recommended range. For fats, the recommendation for adults aged 19 years and older suggests a contribution of 20–35% of total energy intake; the average Thai diet’s fat content of 23.66% corresponds with this recommendation. Protein intake is advised to constitute 10–15% of total daily energy, with an emphasis on consuming both animal- and plant-based protein sources to ensure a complete amino acid profile. Notably, the protein contribution in the average Thai diet was 21.69%, surpassing the DRI recommendation. This may be attributed to the prevalence of protein-rich foods in the Thai diet, such as chicken, eggs, fish, and insects, as illustrated in Table 1. However, the protein intake remained below the cautionary threshold of 30% of total energy, suggesting that it is not considered excessive^24^. The verified average Thai diet was quantitatively assessed to the uptake fluxes of 85 nutrients-derived metabolites, as detailed in Supplementary Table S1.

**Fig. 2:**
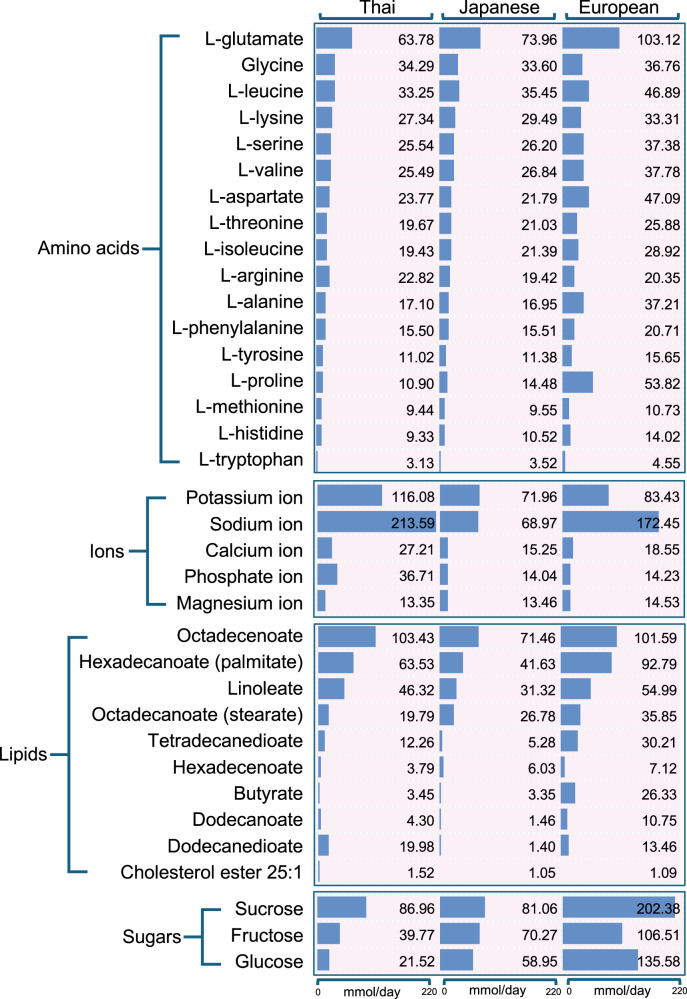
Comparison of nutrients-derived metabolites across average diets of Thai, Japanese, and Europeans. Blue bars represent the uptake flux for each nutrient-derived metabolite (mmol/day), with an axis at the bottom ranging from 0 to 220. The number at the bar’s tip reports the exact value (mmol/day).

Compared to the average diets of the Japanese^25^ and Europeans^23^, 74 out of 85 nutrient-derived metabolites were shared with the Thai diet. Additionally, 35 metabolites classified into amino acids, ions, lipids, and sugars, had an uptake flux exceeding 1 mmol/day across all average diets of Thais, Japanese, and Europeans, as illustrated in Fig. 2. The comparative analysis highlighted distinct dietary patterns among the populations which the Thai diet was characterized by high salt and specific lipids, the European diet featured a wide variety of nutrients, particularly sugars, and the Japanese diet was marked by overall moderation.

### Functional characterization of Thai CSMMs

Metagenomic sequencing data from 86 Thai adults, 31 females and 55 males, aged 18–74 years, with body mass index (BMI) ranging from 18.5 to 30, were used to construct Thai CSMMs. CSMM statistics for 86 Thai adults involving microbiome-level reactions and metabolite abundances listed in Supplementary Table S2. On average, Thai CSMMs contained 107886 ± 17564 metabolic reactions, 95989 ± 15438 metabolites, and 93 ± 15 GSMMs, with a secretion capacity of 226 ± 14 secreted metabolites (Table 2).

**Table 2:** Summary Thai CSMM characteristics

Functional characteristics	Mean ± SD	Min–Max
Number of metabolic reactions	107886 ± 17564	56056–144371
Number of metabolites	95989 ± 15438	48940–127663
Number of GSMMs	93 ± 15	47–126
Secretion capacity (Number of secreted metabolites)	226 ± 14	180–248

**Fig. 3:**
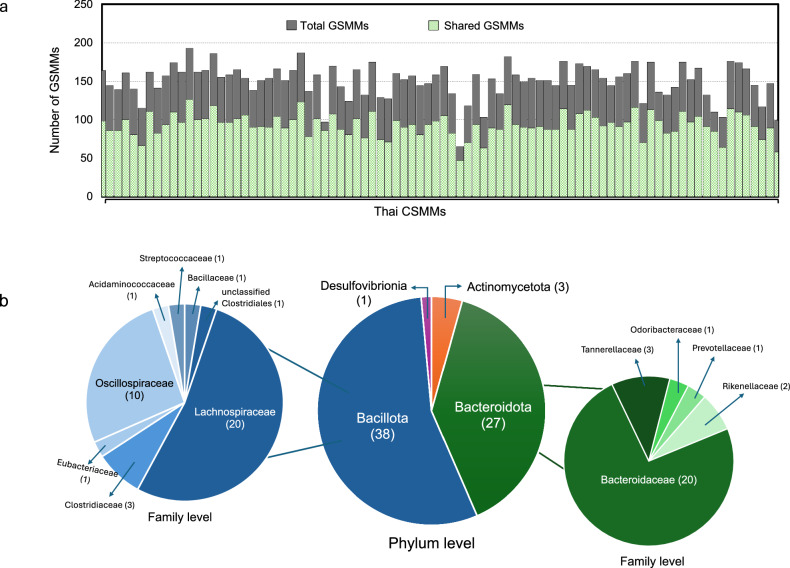
Taxonomic composition of GSMMs in Thai CSMMs. **a** Bar graph shows the total number of GSMMs and the number of shared GSMMs across Thai CSMMs. **b** Pie chart shows the taxonomic distribution of shared GSMMs at the phylum and family levels. Numbers in parentheses indicate the count of shared GSMMs within each lineage.

Regarding the composition of GSMMs in Thai CSMMs, a total of 69 GSMMs were identified as consistently present across the majority of Thai individuals (50% prevalence), as illustrated in Fig. 3a. These shared GSMMs included the four major bacterial phyla, predominantly Bacillota (38 GSMMs) and Bacteroidota (27 GSMMs), with fewer representatives from Actinomycetota (3 GSMMs) and Desulfovibrionia (1 GSMM) as shown in Fig. 3b. Bacillota and Bacteroidota accounted for over 90% of the core GSMMs. At the family level, Lachnospiraceae and Bacteroidaceae were the most represented (20 GSMMs in each family), followed by Oscillospiraceae (10 GSMMs). At the species level, *Faecalibacterium prausnitzii* was the most frequently represented, with four distinct GSMMs detected. Other species included *Mediterraneibacter torques*, *Agathobacter rectalis*, and *Bacteroides thetaiotaomicron*, each represented by two GSMMs (Supplementary Table S3).

Simulations of Thai CSMMs under average Thai dietary constraints, the secretion capacity ranged from 180 to 248 secreted metabolites per individual. A comparable distribution for secreted metabolites is provided in Supplementary Table S4. Briefly, core fermentation has identified by-products, such as carbon dioxide, ammonia, water, and protons which were most abundant. Nucleobases, nucleosides, polyamines, and amino acids were broadly secreted, alongside lactic acid, ethanol, and hydrogen. Notably, SCFAs including acetate, propionate, butyrate, and isobutyrate were consistently produced across all Thai CSMMs (Table 3). While acetate, propionate, and butyrate exhibited relatively low inter-individual variability, a branched SCFA, e.g., isobutyrate, demonstrated significant variability with some individuals showing markedly low secretion levels (below 1 mmol/day). These results highlight the individualized nature of branched SCFA production and its potential implications for personalized health strategies.

**Table 3:** Secretion metabolic fluxes of SCFAs

SCFAs	Flux value (mmol/day)	
	Mean ± SD	Min–Max
Acetate	385.03 ± 6.09	367.19–396.39
Propionate	229.37 ± 49.71	112.83–362.10
Butyrate	113.50 ± 49.79	9.54–272.26
Isobutyrate	77.63 ± 52.16	0.48–208.79

**Fig. 4:**
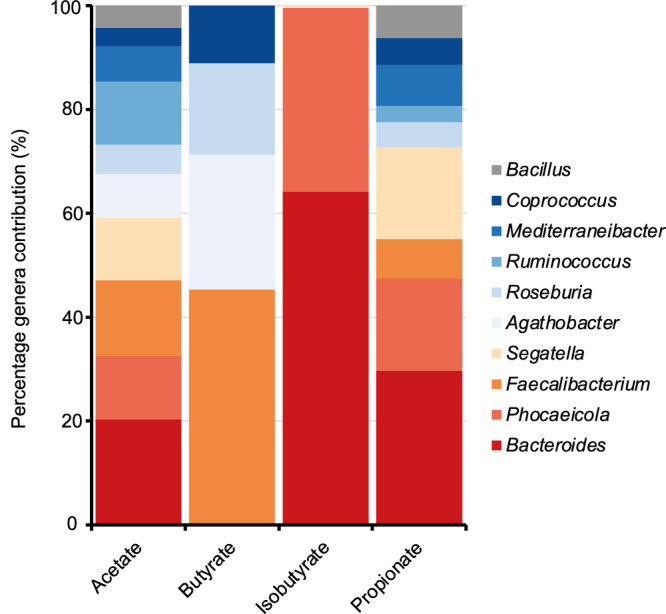
A comparable contribution of gut microbial genera to the secretion fluxes of SCFAs.

Given the essential roles of SCFAs in host physiological functions, a targeted analysis was conducted to identify the genera contributing to SCFA secretion fluxes in Thai CSMMs. The analysis revealed 119 genera contributing in the SCFA secretion fluxes, each with an average of at least 0.01 mmol/day for at least one SCFA, as detailed in Supplementary Table S5. Figure 4 highlights the top 10 genera exhibiting the highest total SCFA secretion fluxes. Among these, *Bacteroides* demonstrated substantial secretion fluxes for acetate, isobutyrate, and propionate. *Phocaeicola* and *Segatella* showed considerable fluxes for propionate, with *Phocaeicola* additionally contributing to isobutyrate. *Faecalibacterium* were prominently contributions to acetate and butyrate secretion, while *Agathobacter, Coprococcus,* and *Roseburia* were associated with butyrate secretion. Propionate secretion was shared among microbial taxa, with notable contributions from *Mediterraneibacter*, *Bacillus*, and *Ruminococcus*. Noticeably, isobutyrate secretion was more restricted, with *Bacteroides* and *Phocaeicola*.

### Modeling of gut microbiome responses to prebiotic intervention

To further demonstrate the utility of CSMMs for studying the impact of diet on gut microbial metabolism, we also applied this approach to investigate the effects of prebiotic intervention in healthy Thai adults. In this study, we focused on manno-oligosaccharides (MOS) derived from copra meal hydrolysate (CMH), a coconut by-product abundantly produced in Thailand. MOS was selected because MOS supplementation can improve gastrointestinal symptoms^26^. Furthermore, integrative metagenomic analysis has highlighted the association of MOS fermentation with SCFA metabolism^6^. However, the specific microbial taxa responsible for these prebiotic effects remain unclear. To address this, we employed CSMMs with shotgun metagenomic data generated in a previous study^6^. The CSMMs were subsequently simulated under MOS- and placebo-supplemented conditions. The analysis of microbial contributions to target metabolites was further applied to identify potential microbial responders to prebiotic intervention by identifying genera exhibiting uptake fluxes for MOS. The CSMM simulations revealed 8 genera responding to prebiotic intervention, each showing MOS uptake fluxes averaging at least 1 mmol/day across all models, as detailed in Table 4. Among the key findings, *Bifidobacterium* demonstrated the most prominent and consistent response, contributing significantly to MOS uptake. Other genera with contributions to MOS uptake included *Agathobacter, Subdoligranulum, Mediterraneibacter,* and *Faecalibacterium*. Overall, *Bifidobacterium* emerged as a predominant prebiotic-responsive genus in fermenting complex carbohydrates, such as MOS.

**Table 4:** The list of microbial responders to prebiotic intervention and their uptake fluxes of MOS

Microbial genera	Uptake fluxes of MOS (mmol/day)	
	Mean ± SD	Min–Max
*Bifidobacterium*	12.58 ± 2.30	9.69–15.28
*Agathobacter*	4.79 ± 0.39	4.27–5.11
*Subdoligranulum*	2.41 ± 0.22	2.00–2.55
*Mediterraneibacter*	3.26 ± 1.10	1.82–5.11
*Faecalibacterium*	4.44 ± 1.34	1.76–5.11
*Coprococcus*	2.64 ± 1.35	1.44–5.11
*Dorea*	2.07 ± 0.63	0.95–2.55
*Blautia*	1.10 ± 0.56	0.30–1.84

## Discussion

Diet plays a central role in shaping gut microbiome composition and metabolic activity, yet assessing gut microbiome responses to dietary interventions remains challenging due to the complexity of host-microbe interactions. While metabolic modeling offers a promising tool for integrating dietary and gut microbiome data^27^, it still faces significant gaps in Thai population. In this study, we developed an integrative modeling pipeline to simulate the effects of diet and prebiotic interventions on gut microbial metabolism. This approach integrated national dietary intake data with GSMMs of the human gut microbiome, toward a systems-level exploration of diet-gut microbiome interactions in Thai population.

Given the unique characteristics of the Thai diet, an average Thai diet was constructed by integrating national FCS^21^ with the Diet Designer tool^23^, supplemented with manual calculations for native Thai food items using the Thai FCD^22^. The macronutrient distribution of carbohydrates and fats in the constructed average Thai diet was consistent with the recommended ranges for the general Thai population^24^. Although protein intake in the average Thai diet remained below the 30% threshold, it slightly exceeded the Thai DRI recommendation (10–15%), consistent with reports of Thai urban sedentary workers consuming over 15% of energy from protein^28^. As opposed to carbohydrates and lipids, proteins serve not only as an energy source, but also provide essential amino acids for structural and physiological functions. This observation highlights a shifting trend in protein consumption in Southeast Asia, particularly Thailand^29^, where a wide range of alternative protein sources, including edible insects, contribute significantly to overall energy intake^30^. In comparison with Japanese and Europeans diets, an average Thai diet was notably characterized by a higher sodium level (Fig. 2). This result was consistent with Thai culinary traditions, which are deeply rooted in the use of high-sodium condiments and seasonings. A previous study indicated that approximately 90% of dietary sodium in Thai population comes from condiments, such as fish sauce, soy sauce, salt, and seasoning cubes^31^. Additionally, traditional seasonings, for instances fermented fish products (e.g., nam pla and pla ra), and shrimp paste (kapi) contribute significantly to the overall sodium content in Thai cuisine^32^. While sodium is an integral part of Thai cooking and an indication of a cultural preference for salty flavors, excessive intake has been strongly associated with elevated blood pressure and increased risk of cardiovascular disease^33^. The high sodium level observed in the constructed average Thai diet highlights the importance of monitoring diet quality to inform effective public health strategies^34,35^.

Alongside, Thai CSMMs were developed, which revealed substantial inter-individual variability in both microbial diversity and metabolic capacity. Thai CSMMs captured a representative range of microbial diversity, with an average of 93 ± 15 GSMMs per individual, consistent with the CSMMs previously developed for Western and East Asian populations^17,18,25^. Analysis of shared GSMMs across Thai CSMMs revealed a core microbiome dominated by Bacillota and Bacteroidota, consistent with global reports in healthy gut microbiome^36,37^ and previous findings in Thai adults^8,38^. *Faecalibacterium*, a key gut health biomarker^39^, was detected with multiple GSMMs within the Thai CSMMs, supporting the presence of a robust core microbiome. Furthermore, the presence of *Ruminococcus* and *Bacteroides*, both known for their ability to degrade complex polysaccharides, suggested Thai gut microbiome well-adapted to fiber-rich foods^20,40^.

Moreover, the gut microbiome is increasingly recognized as a vital metabolic organ that facilitates host metabolism and enhances energy as well as nutrient retention through the exchange of secreted metabolites among microbial members and host cells^41,42^. Of which, the microbial-derived SCFAs are particularly well-studied with essential roles in host physiological processes, including immune modulation, energy homeostasis, and maintenance of gut barrier function^43,44^. In this study, the simulated Thai CSMMs under an average Thai diet revealed SCFAs were among the most consistently secreted metabolites. Notably, acetate, propionate, and butyrate, which are the most abundant SCFAs in the human colon and feces, account for 95% of total SCFAs^45^. In contrast, a branched SCFA, i.e., isobutyrate, exhibited markedly higher inter-individual variability. Acetate, propionate, and butyrate were associated with diverse genera, such as *Faecalibacterium*, *Agathobacter*, *Coprococcus*, and *Roseburia*, indicating that overlapping of metabolic functions supports consistent SCFAs production^41,42^. On the other hand, isobutyrate secretion was limited to *Bacteroides* and *Phocaeicola*, with variation in *Bacteroides* metabolic capacity potentially contributing to differences in branched SCFAs levels^46^. Branched SCFAs are primarily produced by the fermentation of branched-chain amino acids, especially valine and leucine, by specific protein-fermenting bacteria in the gut^47,48^. Our findings suggest that branched SCFAs metabolism is personalized, influenced by diet and gut microbiome composition. Beyond the effects of slow colonic transit and elevated fecal pH, which shift microbial activity from saccharolysis towards proteolysis^49^, dietary protein intake plays a central role. When consumed in excess, protein delivers surplus nitrogen to the colon, thereby accelerating microbial protein fermentation and generating metabolites, such as branched SCFAs, ammonia, indoles, and hydrogen sulfide^50^. These proteolytic products have been implicated in barrier dysfunction, inflammatory responses, and an increased risk of colorectal cancer^51^. Because branched SCFAs derived from the fermentation of branched-chain amino acids. Measurement of fecal branched SCFAs provides a non-invasive marker of colonic protein fermentation, enabling monitoring of dietary protein-microbiome interactions and supporting personalized strategies to mitigate the adverse effects of excessive proteolysis.

Advancing microbiome modulation interventions, especially with prebiotics, has emerged as a promising strategy for improving human health within a broader approach to lifestyle and wellness^52,53^. However, the microbial responses to prebiotics vary considerably, with many clinical trials reporting a high proportion of non-responders^54,55^, although those responders often share specific carbohydrate-degradative systems, which are essential for their utilization. This complication makes identifying microbial responders remain a challenge. To address this, the present study used an integrated diet-gut microbiome modeling pipeline to investigate microbial responses to prebiotics, focusing on the contribution of microbial taxa to target metabolites under simulated gut conditions using copra meal hydrolysate (CMH)^56^. According to nutritional profiling, mannotriose, a type of MOS, was identified as a predominant prebiotic component in CMH^57^. Among the taxa analyzed, *Bifidobacterium* consistently exhibited the most pronounced and widespread metabolic response to the uptake of prebiotic-derived MOS (Table 4). This genus showed high metabolic engagement with prebiotic intervention. A randomized trial found no significant increase in *Bifidobacterium* abundance after CMH intervention, but a marked enrichment of Bifidobacteriaceae occurred during the washout period^26^. These results provide informed suggestions derived from integrated diet-gut microbiome modeling in assessing microbial responses to prebiotics. Additionally, *Bifidobacterium* emerges as a key target genus for precision prebiotic strategies, particularly those using functional ingredients like CMH to support gut health through microbiome modulation. Nevertheless, human studies on MOS derived from CMH remain challenges, especially those assessing gut microbiome outcomes. A larger randomized trials with longitudinal and multi-omics designs are needed to determine the MOS-driven microbiome shifts. In addition, modeling MOS-microbiome interactions in the present study offers a promising path to systematically evaluate other prebiotics. Specifically, future studies should integrate CSMMs with other prebiotics to define responder phenotypes and inform precision prebiotic recommendations.

Moreover, while our work represents the first step in adapting CSMMs to the Thai diet by integrating the Thai-specific dietary profile to provide insights into diet-host-microbiome interactions in this underrepresented population, several limitations should be acknowledged. First, current CSMM repositories remain predominated by isolates from Western cohorts, which restricts their applicability to Southeast Asian populations. The current study underlines the importance of future work in cultivating and sequencing Thai-derived isolates for incorporation into the next-generation CSMMs. Second, although SCFAs production by common gut microbes has been studied extensively, our contribution lies in demonstrating how diet-specific inputs reshape metabolite fluxes, systematically benchmarking these outputs against existing literature, and revealing gaps in the representation of the Southeast Asian microbiome within current model databases. Finally, the prebiotic analysis should be viewed as a proof-of-concept application, illustrating how the Thai CSMM framework can be leveraged to test dietary interventions. Future studies will be explored to validate these model outputs using longitudinal, multi-omics, and clinical trial data to ensure the model’s relevance into practical applications for precision nutrition.

Overall, this study introduces a novel systems-based modeling pipeline to assess microbial responses to diet and prebiotics in the Thai population. By integrating gut microbiome profiles with population-specific dietary data, the CSMMs advances gut microbiome and precision nutrition research. The model-informed suggestions provide valuable insights to support the development of data-driven, tailored dietary strategies for improving health outcomes through diet-gut microbiome interactions.

## Methods

### Quantitative calculation of metabolite constraints for constructing an average Thai diet

An average Thai diet was defined based on food consumption data collected in Thailand from 2013 to 2015 through a survey of 8478 individuals^21^. The analysis focused on estimating the average intake (g/day) of the most commonly consumed food items among Thais over the age of three. Using these intake values, each food item was identified and matched with data from the VMH database to calculate the uptake flux of nutrient components in mmol/day^23^. When an exact match was unavailable, the closest related food entries were used. However, certain traditional Thai foods lacked nutrient composition data in the VMH database. To address this, nutrient component information for these specific foods was retrieved from the Thai FCD^22^ and manually calculated their uptake flux values. The uptake fluxes obtained from both the VMH Diet Designer and manual calculations were then combined to determine the overall uptake flux of nutrient components. This final dataset serves as the dietary constraints for simulating CSMMs tailored to the Thai population.

### Metagenomic sequencing data analysis and taxonomy assignment for constructing Thai CSMMs

Paired-end Illumina raw reads of healthy Thai adults were obtained from the Sequence Read Archive (www.ncbi.nlm.nih.gov/sra; accession date: 30/1/2025) from the SRA BioProjects: the TIGER-LC cohort published by Pomyen et al., which included 76 samples (PRJNA932948)^58^ and the Thai gut microbiome meta-gene catalog published by Raethong et al. included 10 samples (PRJNA637175)^8^. For the pre-processing process, the reads were assessed for quality scores across all bases using the FastQC analysis tool. The metagenomic reads for each file were trimmed using BBDuk (BBTools version 38.9) with the default parameters. Subsequently, to remove human contaminant sequences, the reads were aligned to the human genome version GRCh38 using BBMap with default parameters. Reads mapping to the host genome were discarded, and unmapped reads were retained.

For the taxonomy assignment, the pre-processed reads were mapped to a reference set of 773 genomes of the assembly of gut organisms through reconstruction and analysis (AGORA) version 1.03 (published on 25.02.2019, available at https://www.vmh.life)^13^ using CoverM contig (Galaxy Version 0.7.0, Freiburg Galaxy Team)^59^. Before mapping, the genomes were concatenated into one file, where each was represented as an individual chromosome of the AGORA model identifier. Mapping was performed using BWA-MEM with default parameters^60^. Genome coverage, defined as the number of mapped reads normalized by genome size, was calculated by CoverM, which generated tab-delimited output files containing AGORA model identifiers that related to the GSMMs in AGORA version 1.03 and corresponding abundance values for each sample. The assigned AGORA model identifiers and their abundances were subsequently merged into a single summary table using an in-house MATLAB script, which is publicly available at the GitHub repository (https://github.com/nachonase/ThaiCSMMs). A single summary table of AGORA model identifiers with their abundances was the normalized using the *normalizeCoverage* function from the Microbiome Modeling Toolbox 2.0^14,15^, applying a cut-off of >0.001%. The resulting list of AGORA model identifiers was then assigned to taxonomic ranks and thoroughly cross-checked against the NCBI taxonomy database (https://www.ncbi.nlm.nih.gov/datasets/taxonomy; accession date: 23/9/2025). The final list of taxonomically assigned AGORA model identifiers with their relative abundances is provided in supplementary Table S6.

### Construction of Thai CSMMs and modeling response to an average Thai diet

The simulations of CSMMs were performed in MATLAB version R2024a (MathWorks, Inc.) as the programming environment, using the IBM CPLEX solver (version 128) (IBM, Inc.) for model optimization. These simulations utilized functions from the Microbiome Modeling Toolbox 2.0^14,15^, which is a part of COBRA Toolbox^61^. Codes and models developed and used in this study are available at the GitHub repository (github.com/nachonase/ThaiCSMMs).

The CSMMs were constructed using a reference set of 773 GSMMs from AGORA version 1.03 (published on 25.02.2019, available at www.vmh.life; accession date: 24/1/2025)^13^. The final list of AGORA model identifiers with their abundances was used as input, and GSMMs with relative abundances greater than 0.001% were included in the CSMMs. The flux through each GSMM was coupled to its biomass objective function, and the microbiome metabolic biomass reaction was parameterized by applying relative abundances of the assigned AGORA model identifiers as stoichiometric values. These constraints ensured that the GSMMs grew at experimentally measured ratios. The allowed flux through the microbiome metabolic biomass reaction was set between 0.4 and 1 mmol/day, corresponding to fecal emptying every three days to once per day^17^. The CSMMs were then simulated the response of gut microbiome metabolism to an average Thai diet using FVA. The overall construction process and modeling were run using *initMgPipe* function from the Microbiome Modeling Toolbox 2.0^14,15^. The net uptake was calculated as the sum of maximal uptake and minimal secretion, while the net secretion was determined by summing maximal secretion and minimal uptake. The contributions of microbial taxon to target metabolites were identified and quantified. Unless otherwise specified, all calculations were performed using the default software parameters.

### Modeling gut microbiome response to prebiotic intervention

Shotgun metagenomic reads from the Sequence Read Archive (www.ncbi.nlm.nih.gov/sra; accession date: 30/1/2025) from the SRA BioProject PRJNA596624 published by Kingkaw et al. were obtained and used to construct the CSMMs for investigating prebiotic responders^6^. The construction process followed the same procedure as the Thai CSMMs, except that dietary constraints were defined to simulate gut microbiome metabolism under MOS supplementation or placebo. The doses of MOS and placebo were translated from the intervention study by Kingkaw et al., in which the placebo group consumed 10 g of maltodextrin per day, while the prebiotic group received 5 g of MOS plus 5 g of maltodextrin per day^6^. For the placebo condition, 10 g of maltodextrin was converted into mmol equivalents and incorporated into the average Thai diet. For the prebiotic condition, the nutrient composition of MOS was derived from published data^57^, combined with 5 g of maltodextrin, and added to the average Thai diet. The contributions of microbial taxon to target metabolites analysis were applied to identify the microbial responders to prebiotic intervention using *predictMicrobeContributions* function from the Microbiome Modeling Toolbox 2.0^14,15^.

This study used de-identified human gut microbiome data from public databases; no new human participants were recruited. The study protocol No. MU-CIRB 2022/095.0704 was approved by the Mahidol University Central Institutional Review Board (MU-CIRB) with Certificate of Exemption No. MU-CIRB 2022/061.0605.

## Supplementary information

Supplementary materials

## Data Availability

The sequencing data used in the project are openly available from SRA. Raw data supporting the findings (e.g., nutrient-derived metabolites in the average Thai diet and CSMM statistics for Thai adults) are provided as Supplementary Material.

## Code availability

Codes and models developed and used in this study are available at https://github.com/nachonase/ThaiCSMMs.
